# 

**Published:** 1859-08

**Authors:** 


### RECEIPTS FOR THE JOURNAL, UP TO AUGUST 2, 1859.

**ILLINOIS.**

| | | |
|---|---|---|
| Dr. G. H. Kittell, | vol. 2, 1859, | $2 |
| " C. N. Camp, | " " | 2 |
| " Wm. McKnight, | ½ " " | 1 |
| " B. Harris, | " " | 2 |
| " Wickersham, | " " | 2 |
| " R. H. Paddock, | " " | 2 |
| " Matthei, | " " | 2 |
| " T. D. Fitch, | vol. 1, 1858, | 2 |
| " Geo. W. Hewitt, | vols, 1 & 2, 1858–9, | 4 |

**INDIANA.**

| | | |
|---|---|---|
| Dr. Geo. P. Nicolai, | vol. 2, 1859, | $2 |

**WISCONSIN.**

| | | |
|---|---|---|
| Dr. Clark Nettleton, | vol. 2, 1859, | $2 |

**IOWA.**

| | | |
|---|---|---|
| Dr. W. S. Robertson, | vol. 2, 1859, | $2 |

**CALIFORNIA.**

| | | |
|---|---|---|
| Dr. D. Ray, | vol. 2, 1859, | $2 |

